# Lipopolysaccharide alters decorin and biglycan synthesis in rat alveolar bone osteoblasts: consequences for bone repair during periodontal disease

**DOI:** 10.1111/j.1600-0722.2008.00535.x

**Published:** 2008-06

**Authors:** Helen C Roberts, Ryan Moseley, Alastair J Sloan, Sarah J Youde, Rachel J Waddington

**Affiliations:** Tissue Engineering and Reparative Dentistry, School of Dentistry, Cardiff University, Wales College of MedicineHeath Park, Cardiff, UK

**Keywords:** alveolar bone, lipopolysaccharide, osteoblasts, *Porphyromonas gingivalis*, proteoglycans

## Abstract

A prime pathogenic agent associated with periodontitis is lipopolysaccharide (LPS) derived from *Porphyromonas gingivalis*. This study investigated the effects of *P. gingivalis* LPS on osteoblasts, which are responsible for alveolar bone repair. Bone cells were obtained from explants of rat alveolar bone chips and cultured with 0–200 ng ml^−1^ of *P. gingivalis* LPS. *Porphyromonas gingivalis* LPS significantly increased cell proliferation and inhibited osteoblast differentiation, as judged by reduced alkaline phosphatase activity. Analysis of biglycan mRNA and protein levels indicated that *P. gingivalis* LPS significantly delayed the normally high expression of biglycan during the early stages of culture, which are associated with cell proliferation and early differentiation of progenitor cells. In the presence of *P. gingivalis* LPS, decorin expression by the alveolar bone cells was reduced during periods of culture relating to collagen fibrillogenesis and mineral deposition. Analysis of glycosaminoglycan chains conjugated to these proteoglycans suggested that in the presence of *P. gingivalis* LPS, dermatan sulfate persisted within the matrix. This study suggests that *P. gingivalis* LPS influences the expression and processing of decorin and biglycan in the matrix, altering alveolar bone cell activity and osteoblast phenotype development. The consequences of this altered expression in relation to hindering bone repair as part of the cycle of events during periodontal disease are discussed.

Almost all forms of periodontal disease occur as a result of mixed microbial infections. However, intensive studies have suggested a prime pathogenic role for *Porphyromonas gingivalis* (a gram-negative anaerobic bacterium) and its constituent lipopolysaccharide (LPS) that is present on the *P. gingivalis* outer membrane (reviewed in Ref. 1). The pathogenic action of *P. gingivalis* LPS leading to periodontal tissue destruction is unclear, possibly because its action is multifactorial and affects many cell types present within the periodontium. Several studies have demonstrated that *P. gingivalis* LPS promotes the expression of pro-inflammatory cytokines and chemokines in monocytes and macrophages (2), dendritic cells (3), and the resident gingival connective tissue fibroblasts (4). Pertinent to inflammatory alveolar bone destruction, *P. gingivalis* LPS also increases the release of inflammatory osteolytic factors from osteoblastic cells to stimulate, indirectly and directly, osteoclastic bone resorption (5–9).

The pathology of inflammatory periodontal disease progresses through cycles of tissue destruction, followed by periods of quiescence and tissue repair. The severity of tissue damage may therefore be considered to be directly proportional to the induction of the host immuno-inflammatory response and the inability of the periodontal tissues to undergo repair. During wound healing, bone tissue repair is facilitated by the recruitment of progenitor cells that are present in the periodontal ligament adjacent to the alveolar bone and the bone marrow space. A cocktail of growth factors, such as bone morphogenic proteins (BMP), transforming growth factor-β (TGF-β), and platelet derived growth factor (PDGF), which are released as part of the repair process, stimulate the proliferation and differentiation of the progenitor cells to form osteoblasts that are capable of the eventual synthesis of a mineralized matrix (10, 11).

In the repair process, the structural proteins of the extracellular matrix play major roles in the formation of a functional tissue. Important proteins in this process are the proteoglycans decorin and biglycan, which are characterized by a common 45–50 kDa leucine-rich repeat core protein, the N-terminal of which is conjugated to one and two glycosaminoglycan (GAG) chains, respectively (12, 13). Both decorin and biglycan are present in significant amounts in mineralized tissues, where the GAG chains are predominantly chondroitin sulfate (CS) (14, 15). By contrast, in non-mineralized connective tissues, such as skin, ligament and tendon, dermatan sulfate (DS) GAG chains predominate (16), although such forms of decorin and biglycan have also recently been found in the premineralized tissues of osteoid and predentine (17, 18). Through their differential expression during osteogenesis (17) and their varied affinities with matrix components, decorin and biglycan exert different effects on collagen fibrillogenesis (19–22), on the presentation of growth factors, such as TGF-β (23, 24) and tumor necrosis factor-α (TNF-α) (25), and in the regulation of hydroxyapatite crystal growth and morphogenesis (22, 26, 27). It is being increasingly recognized that the tempero-spatial expression of the various structural and signalling components during bone remodelling is not only important in determining tissue repair leading to healthy tissue regeneration, but is critical in the matrix’s influence of events associated with tissue damage (10).

Although the influence of LPS on osteoclasts and bone resorption has been studied extensively (9, 28–30), the underlying effects of *P. gingivalis* LPS on osteoblasts and the bone repair process are less well defined. Some studies have indicated that *P. gingivalis* LPS and sonicated bacterial extracts inhibit various osteogenic parameters, such as alkaline phosphatase activity, collagen synthesis and bone nodule formation, implying that periodontal pathogens inhibit bone formation (5, 31–33). A limited number of studies have further investigated the effects of *P. gingivalis* LPS on the production of extracellular matrix protein associated with bone formation. Kadono*et al.* (33) demonstrated that *P. gingivalis* LPS inhibited the production of osteocalcin and osteopontin in rat calvaria cells. However, the influence of *P. gingivalis* LPS on other matrix proteins is poorly understood.

The present study aimed to investigate the effects of *P. gingivalis* LPS extract on osteoblastic proliferation and phenotypic development leading to the synthesis of a mineralized matrix, using a novel *in vitro* alveolar bone culture system. The use of primary osteoblasts, derived from alveolar bone, provides a significant model culture system that is highly representative for the study of inflammatory periodontal disease. Past studies have indicated that alveolar bone, compared with other osseous sites, has a high metabolic turnover rate, has compositional differences within the extracellular matrix, and is strongly influenced by the cellular activity of the periodontal ligament in contributing progenitor cells (15, 34–36), which may reflect its increased susceptibility to periodontal disease.

## Material and methods

### Isolation and culture of alveolar bone cells

Alveolar bone immediately surrounding incisor and molar teeth was dissected from 28-d-old male Wistar rats. Bone fragments were cleared of periodontal ligament by treatment with 1 mg ml^−1^ of type IV collagenase/4.5 U ml^−1^ of type IV elastase (Sigma-Aldrich, Gillingham, UK) for 2 h at 37°C and then washed several times with sterile phosphate-buffered saline (PBS). The bone fragments were cultured in RPMI-1640 and F-12 HAMS (1:1) containing 10% fetal bovine serum (heat inactivated) (Invitrogen, Paisley, UK), 5 mg ml^−1^ of transferrin-sodium-selenite (Sigma-Aldrich), and antibiotics (10,000 units ml^−1^ of penicillin G, 10 mg ml^−1^ of streptomycin, and 25 mg ml^−1^ of amphotericin B) (Invitrogen), at 37°C in an atmosphere of 5% CO_2_. Initial cell migration from the bone fragments appeared at 5–7 d, with confluency around 3–5 wk, after which cells were reseeded at 1 × 10^4^ cells cm^−2^ and cultured in the above medium. After 5 d in culture, the cells were supplemented with 50 *μ*g ml^−1^ of ascorbic acid, 10 mM β-glycerophosphate, 10^−8^ M dexamethasone, and 1 mM CaCl_2_ (Sigma-Aldrich). In developing this protocol, various types of culture media were investigated to identify the optimal growth conditions. Of note, cells incubated in the presence of mineral-inducing factors, 10 mM β-glycerophosphate, 10^−8^ M dexamethasone, and 50 *μ*g ml^−1^ ascorbic acid, from the onset of culture, maintained poor cell viability and thus failed to develop an osteoblastic phenotype or to produce mineralizing bone nodules.

Phases relating to cell growth, matrix synthesis, and mineral deposition were determined for the duration of the culture. Viable cell counts were determined every 24 h during 1–5 d of culture, by staining with 0.5 mg ml^−1^ of ethidium bromide/0.15 mg ml^−1^ of acridine orange and viewing immediately under a fluorescence microscope. Matrix synthesis was monitored over 5–28 d of culture. Cells were fixed with 2% formaldehyde for 30 min and then blocked with normal horse serum (Sigma-Aldrich) for 10 min. Cells were incubated for 1 h with rabbit monoclonal anti-human collagen type I (Biodesign International, Saco, ME, USA; 1:10), rabbit polyclonal to anti-rat osteocalcin (Cambio, Cambridge UK; 1:50), anti-human osteopontin (LF123; 1:50), anti-mouse osteonectin (LF-23; 1:50), or human bone sialoprotein (LF28; 1:50) (LF antibodies from Dr L. Fisher, NIH, Bethesda, MD, USA). All antibodies used had previously been reported to exhibit cross-reactivity with rat tissues. Immunoreactivity was detected using the universal peroxidase Vectastain (Vector Laboratories, Burlingame, CA, USA). Negative controls included the omission of primary antibodies. The presence of calcium phosphate mineral deposition was determined by von Kossa staining (37).

### Preparation of *Porphomonas gingivalis* LPS

*Porphomonas gingivalis* W50 was cultured anaerobically in fastidious anaerobic broth (Lab M, Bury, UK) for 72 h, 37°C. Lipopolysaccharide was extracted from bacterial cells using the hot phenol–water method described by Westphal & Jann (38). Briefly, pelleted bacterial cells were suspended in equal volumes of 90% phenol and double-distilled water, shaken vigorously for 20 min at 65–68°C, and immediately cooled in an ice bath. Following centrifugation at 8,000 ***g*** for 20 min, the upper aqueous layer, containing the crude LPS, was collected. The remaining insoluble and phenol layers were re-extracted further with double-distilled water and pooled with the above collected aqueous extract. Aqueous extracts were exhaustively dialysed against double-distilled water and then lyophilized. The extract was treated with 2% cetyltrimethylammonium bromide (Sigma-Aldrich) for 15 min at room temperature to precipitate nucleic acids, centrifuged at 3,000 ***g*** for 20 min, and the collected supernatant was then lyophilized. To remove contaminating bacterial protein, the LPS extract was resuspended in 0.5 M sodium chloride and incubated with a 10-fold greater volume of ethanol for 2 h at 4°C. Following centrifugation at 8,000 ***g*** for 20 min, the supernatant was collected and lyophilized. To provide characterization, LPS was resuspended in sample buffer [0.062 M Tris–HCl, pH 6.8, 10% glycerol, 2% sodium dodecyl sulphate (SDS), 5% 2-mercaptoethanol, 0.002% Bromophenol blue) and separated by electrophoresis on 4–15% preformed polyacrylamide gels within a Phastsystem (GE Healthcare, Giles, UK). Lipopolysaccharide was detected based on the silver staining method of Tsai & Frasch (39), using the PlusOne silver staining kit (GE Healthcare). Removal of contaminating proteins was confirmed by staining SDS–polyacrylamide gels with 0.05% Coomassie Brilliant Blue, 50% methanol, 10% acetic acid for 1 h and destaining with 50% methanol, 10% acetic acid. The absence of nucleic acids was determined by electrophoresis of samples on 2% agarose gels containing 50 ng ml^−1^ of ethidium bromide and using a Tris-Borate-EDTA (TBE) buffer system (Sigma-Aldrich), followed by visualization under ultraviolet (UV) light using the BioRad image analysis system (BioRad Laboratories, Hemel Hempstead, UK).

### Cell cytotoxicity assay

Alveolar bone cells, seeded at a density of 1 × 10^4^ cells cm^−2^ in six-well plates, were cultured in the starting medium described above, in the presence of 0–1 *μ*g ml^−1^ of *P. gingivalis* LPS. After 24 h, cells were stained with 0.4% Trypan blue (Sigma-Aldrich). Blue-staining non-viable cells, and non-staining viable cells, were immediately counted under an inverted microscope and the percentage viability was determined. Assays were performed in triplicate. Cells incubated in the presence of 0, 100, and 200 ng ml^−1^ of LPS demonstrated viability counts of between 95 and 98% and therefore were used in subsequent analyses to determine the non-cytotoxic effects of LPS on alveolar bone cells.

### Influence of LPS on cell behaviour

Alveolar bone cells were reseeded into six-well plates at 1 × 10^4^ cells cm^−2^ and cultured with 2 ml of medium, as described above, supplemented with 0, 100 or 200 ng ml^−1^ of *P. gingivalis* LPS. At 1–5 d, 400 *μ*l of medium was removed from triplicate wells at each of the LPS concentrations, replaced with 400 *μ*l of 5 mg ml^−1^ MTT formazan (Sigma-Aldrich), and incubated at 37°C in a 5% CO_2_ atmosphere for 4 h, to allow uptake of the dye by viable cells. The MTT-containing medium was removed and the cells were washed with PBS and lysed with 1 ml of 0.5 M dimethylformamide, 20% SDS, at 37°C in a 5% CO_2_ atmosphere for 4 h. The amount of dye released was quantified at 570 nm.

Similarly, alveolar bone cells, cultured in six-well plates at 1 × 10^4^ cells cm^−2^ in 2 ml of medium, supplemented with 0, 100 or 200 ng ml^−1^ of *P. gingivalis* LPS, were examined for membrane-bound alkaline phosphatase activity as a marker for osteoblast differentiation. Over 2–28 d, cells from triplicate wells at each experimental condition were treated with 0.1% Triton X-100, 50 mM Tris–HCl buffer, pH 7.2, for 15 min at 37°C. Membrane-released proteins were desalted using PD-10 columns (GE Healthcare) and alkaline phosphatase activity was determined by measuring the hydrolysis of *p*-nitrophenol using a commercial kit (ALP Optimized; Sigma-Aldrich). *P*-values were calculated using one-way analysis of variance (anova) with the Turkey post-correction test if the *P*-value was < 0.05 (Instat Package; GraphPad Software, San Diego, CA, USA), to determine the direct differences in cell numbers and alkaline phosphatase activity for LPS-supplemented and unsupplemented cells, at each time point analysed.

### Reverse transcription–polymerase chain reaction

Alveolar bone cells were cultured in six-well plates at 1 × 10^4^ cells cm^−2^ in 2 ml of medium, supplemented with 0 or 200 ng ml^−1^ of *P. gingivalis* LPS, for 28 d. On 2–28 d, cells were washed twice with PBS and the total cellular RNA was extracted using a Quiagen RNeasy Mini kit (Quiagen, Crawley, UK), with contaminating genomic DNA further eliminated using a DNA-free kit (Ambion, Warrington, UK). The quantity and purity of RNA was determined by measuring the absorbance at 260/280 nm, and cDNA was synthesized from 500 ng of total RNA by reverse transcription (Promega, Southampton, UK) using the cycling conditions 25°C for 10 min, 42°C for 60 min, and 95°C for 5 min. Polymerase chain reactions were performed using primer sequences to decorin (sense, 5′-CAATAGCATCACCGTTGTGG-3′; antisense, 5′-CCGGACAGGGTTGCTATAAA-3′; product size 204 bp), biglycan (sense, 5′-CCTCCAGCACCTCTATGCTC-3′; antisense, 5′-ACTTTGAGGATACGGTTGTC-3′; product size 262 bp), and glyceraldehyde-3-phosphate dehydrogenase (GAPDH) (sense, 5′-TCCCTCAAGATTGTCAGCAA-3′; antisense, 5′-AGATCCACAACGGATACATT-3′; product size 307 bp) using the following PCR cycling parameters: one precycle at 94°C for 3 min, followed by 35 cycles at 94°C for 1 min, 58°C for 1 min, 72°C for 1 min, and one final cycle at 72°C for 5 min. Reverse transcription–polymerase chain reaction products were separated by electrophoresis on 2% agarose gels containing ethidium bromide, viewed under UV light using the BioRad image analysis system, and then quantified using the BioRad Quantity One calibration programme (BioRad), to determine band intensities by comparison with GAPDH. Band intensities were measured three times from three separate electrophoretic separations and means and standard deviations were calculated to give a semiquantitative analysis and therefore descriptive, rather than actual, changes in expression levels.

### Purification of proteoglycans synthesized by alveolar bone cells

Alveolar bone cells were reseeded into T-75 flasks at 1 × 10^4^ cells cm^−2^, supplemented with 0 or 200 ng ml^−1^*P. gingivalis* LPS, and cultures maintained for 2–28 d, as described above. Cells were washed twice with PBS and non-collagenous proteins were extracted from the synthesized matrix with 15 ml of 4 M guanidinium chloride, 0.05 M sodium acetate buffer, pH 6.8, containing proteolytic inhibitors (1 mM iodoacetic acid, 5 mM *N*-ethylmalemide, 5 mM benzamidine; Sigma-Aldrich) for 48 h at 4°C, with constant agitation. The guanidinium chloride extracts were exhaustively dialysed, at 4°C, against double-distilled water containing proteolytic inhibitors (described above), followed by dialysis for 1 d against double-distilled water only, and then lyophilized.

Proteoglycans were purified from the non-collagenous extract by anion-exchange chromatography, using a 1-ml Resource Q column incorporated into a fast protein liquid chromatography (FPLC) system (GE Healthcare). Samples were applied to the column in 4 M urea, 50 mM Tris–HCl, pH 6.8 (5 mg ml^−1^). Bound proteins were selectively eluted with a 0–1 M NaCl linear gradient, in the above buffer, over 19 ml. Elution profiles at 280 nm were obtained to identify protein peaks, which were dialysed against double-distilled water and recovered by lyophilization prior to reconstitution at 10 mg ml^−1^ in double-distilled water. Five microlitres of each of the recovered protein fractions was blotted onto nitrocellulose membranes and proteoglycan-rich fractions were identified by their immunoreactivity with antibodies LF113 (anti-decorin) and LF106 (anti-biglycan) (Dr L. Fisher, NIH, Bethesda, MD, USA) using the 5-bromo-4-chloro-3’-indolyphosphate p-toluidine salt (BCIP)/ Nitro Blue tetrazolium (NBT) detection system, described below. Core proteins of the proteoglycans were then obtained for Western blot analysis by digestion with proteinase-free chondroitinase ABC (Seikagaku, Tokyo, Japan), as previously described by Waddington*et al.* (40).

### Western blot analysis

Ten micrograms of core protein samples were dissolved in 30 *μ*l of sample buffer (0.062 M Tris–HCl, pH 6.8, 10% glycerol, 2% SDS, 5% 2-mercapthoethanol, 0.002% Bromophenol blue). Samples were separated (15 mA, for 3 h, at room temperature) on 4–15% precast gradient SDS-polyacrylamide gels (BioRad), using a running buffer of 25 mM Tris–HCl, 0.2 M glycine, 1% SDS. Separated components were electroblotted onto polyvinylidene difluoride (PVDF) membranes (GE Healthcare) in a Trans-Blot SD Semi-Dry Electrophoretic Transfer Cell system (BioRad), for 30 min at 15 V. The membranes were examined for immunoreactivity with polyclonal antibodies LF113 (decorin, 1:100) and LF106 (biglycan, 1:100), visualized using secondary goat anti-rabbit immunoglobulin G (IgG) conjugated to horseradish perioxidase (1:1,000) (Sigma-Aldrich) and the ECL Plus Western Blotting Detection Reagent kit (GE Healthcare), with exposure for 30 min onto Hyperfilm-ECL (GE Healthcare). Images were captured using the BioRad Quantity One image-analysis system (BioRad). As a negative control, to confirm the absence of non-specific binding by the antibodies, primary antibodies LF113 and LF106 were mixed with 100 *μ*g ml^−1^ of their respective immunizing antigen peptide (courtesy of Dr L. Fisher) prior to treatment of protein-blotted PVDF membranes.

### Identification of GAG constituents

Glycosaminoglycan chains were released from the purified proteoglycan samples (0.5 mg ml^**−**1^) with non-specific protease type XIV (Sigma-Aldrich) in 0.2 M Tris–HCl, 10 mM CaCl_2_, pH 7.5, at 55°C, for 18 h, lyophilized, and then resuspended in double-distilled water equivalent to the starting volume of the original sample. Two-microlitre samples were analysed by cellulose acetate electrophoresis to separate the GAG components, as previously described by Waddington*et al.* (40). Glycosaminoglycan components were stained with Alcian blue and identified according to their electrophoretic mobility in relation to a GAG standard (Sigma-Aldrich) and their susceptibility to chondroitinase ABC and chondroitinase AC (40). Staining intensities of the GAG components were measured using the BioRad Quantity One image-analysis system (BioRad Laboratories) and quantified by comparison with the commercial standards.

## Results

### *Porphomonas gingivalis* LPS characterization

Lipopolysaccharide was extracted from the bacterial cell walls using a hot phenol–water differential extraction method. Electrophoretic separation by sodium dodecyl sulphate–polyacrylamide gel electrophoresis (SDS-PAGE), followed by staining with silver nitrate, demonstrated the classical laddering heterogeneous pattern of LPS (data not shown), which is attributable to the variability of the O-specific oligosaccharide moiety (39). Contaminating DNA and proteins were removed by precipitation with cetyltrimethylammonium bromide and ethanol respectively. Staining of SDS-PAGE gels with Coomassie Brilliant Blue indicated successful removal of contaminating bacterial proteins for the preparation from *P. gingivalis*. Similarly, *P. gingivalis* LPS contained minimal nucleic acid content, as assessed by agarose gel electrophoresis followed by staining of the gel with ethidium bromide. For comparison, a sample of commercially available *Escherichiacoli* LPS was also examined by SDS-PAGE and for protein and nucleic acid content. Of, note *E. coli* LPS was shown to contain trace amounts of protein and significant amounts of nucleic acid.

### Characterization of alveolar bone cells in culture

**Fig. 1:**
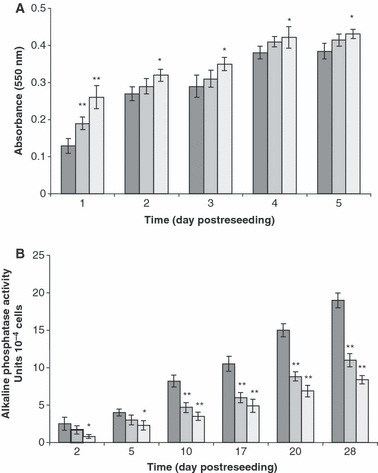
The influence of non-lethal levels of lipopolysaccharide (LPS) on cell behaviour during cell culture. Alveolar osteoblast-like cells were seeded at 1 × 10^4^ cells cm^**−**2^ and cultured in the presence of (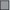) 0 ng ml^**−**1^*P. gingivalis* LPS, (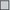) 100 ng ml^**−**1^*P. gingivalis* LPS, or (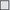) 200 ng ml^**−**1^*P. gingivalis* LPS. (A) Cell numbers were determined for 1–5 d by measuring the uptake by viable cells of the chromagenic substrate, MTT. Viable cell numbers increased in the presence of LPS, compared with the non-supplemented control. This was statistically significant (**P < 0.001) at 1 d for cells in the presence of 100 and 200 ng ml^**−**1^ of LPS and statistically significant (*P < 0.05) at days 2–5 for cells cultured in the presence of 200 ng ml^**−**1^ of LPS. (B) Alkaline phosphatase activity was assessed for cells in culture for 2–28 d. Alkaline phosphatase was released from the cell membrane by treatment with the detergent Triton X-100 and quantified as a measure of p-nitrophenol hydrolysis. For cells cultured in the presence of 200 ng ml^**−**1^ of LPS, alkaline phosphatase was significantly reduced compared with the non-supplemented control (*P < 0.05 and **P < 0.001). Alkaline phosphatase activity was also reduced in the presence of 100 ng ml^**−**1^ of LPS, although this was not statistically significant at 2 and 5 d, but highly significant at 10–28 d (**P < 0.001).

During this study optimal culture conditions were established and cellular activity determined. We found that it was necessary for alveolar bone cells to be cultured first in the absence of mineral-inducing factors. Cell numbers rose sharply over the first 5 d post-reseeding, after which viable cell numbers remained constant (demonstrated in Fig. 1A, no-LPS control). Following cessation of the proliferative stage, mineral-inducing factors (10 mM β-glycerophosphate, 10^**−**8^ M dexamethasone, and 50 *μ*g ml^**−**1^ of ascorbic acid) were added and the cultures were maintained for up to 28 d. Alkaline phosphatase activity was also seen to rise during this period (Fig. 1B, no-LPS control). Over the period of culture (1–28 d), cells were examined, using immunohistochemical techniques, for the appearance of a range of bone protein markers that assisted in defining the start of matrix deposition and confirmed the synthesis of bone-related proteins. von Kossa staining was used to demonstrate the deposition of calcium phosphate mineral salts. For brevity, the results are not shown but described. In order to produce mineralizing nodules, bone cells required a high-calcium environment to promote mineral deposition. Nodules that stained positive for calcium phosphate mineral deposition, as determined using the von Kossa stain, appeared from 17 d post-reseeding. As control studies, dermal fibroblasts were grown in the same high-calcium medium and the lack of mineral deposition around these cells confirmed that mineral formation in the alveolar bone cultures was not a result of spontaneous calcium phosphate precipitation. Immunohistochemical staining of the extracellular matrix demonstrated the presence of collagen type I (from around 7 d post-reseeding), osteopontin (from 10 d), osteonectin (13 d), and bone sialoprotein and osteocalcin (from 17 d). These results thus defined a cell proliferation period over the first 5 d of culture, with osteoid production commencing around 7 d, where collagen synthesis was evident. Calcium phosphate mineral deposition, as evidenced by the detection of bone sialoprotein, osteocalcin and von Kossa staining, was presumed to be established by 17 d. These formed the justification for the analysis of these days throughout the remainder of the study.

### Effect of *P. gingivalis* LPS on cellular behaviour

The influence of *P. gingivalis* LPS on alveolar bone cell numbers, as measured by MTT formazan uptake by viable cells, is shown in Fig. 1A. When compared with cells cultured in the absence of LPS, the presence of 100 or 200 ng ml^**−**1^ of *P. gingivalis* LPS produced a significant, concentration-dependent increase (*P* < 0.05) in cell proliferation over the first 5 d postreseeding. This effect was more pronounced at the initial stages of culture, where cell numbers in the presence of 100 and 200 ng ml^**−**1^ of *P. gingivalis* LPS were increased by 47% and 102% respectively at 1 d (*P* < 0.001). By 5 d, the effect of *P. gingivalis* LPS appeared to be reduced, with cell numbers showing only a 7.4% increase at 100 ng ml^**−**1^ and an 11.1% increase at 200 ng ml^**−**1^ (*P* < 0.05). Figure 1B demonstrates the effect of *P. gingivalis* LPS on membrane-bound alkaline phosphatase activity. Alkaline phosphatase activity was significantly inhibited in cells incubated with *P. gingivalis* LPS, particularly from 10 d onwards (*P* < 0.001). Again, the observed effect was concentration dependent, with activity at 28 d for cells cultured in the presence of 100 ng ml^**−**1^ of LPS decreasing by 42% compared with a decrease of 56% in cells cultured in the presence of 200 ng ml^**−**1^ of LPS.

### Influence of *P. gingivalis* LPS on biglycan and decorin mRNA levels

**Fig. 2:**
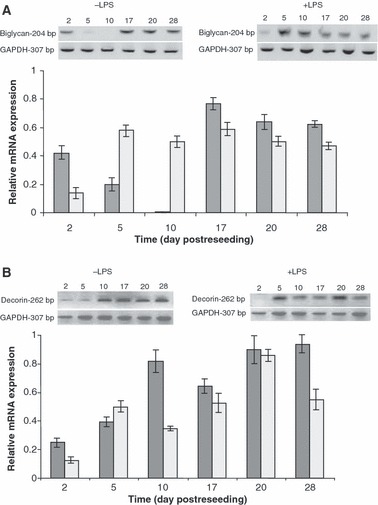
Semiquantitative reverse transcription–polymerase chain reaction (RT-PCR) analysis showing the effect of *Porphyromonas gingivalis* lipopolysaccharide (LPS) on the mRNA levels of (A) biglycan and (B) decorin. Alveolar bone cells were seeded at 1 × 10^4^ cells percm^2^ and cultured in the presence of (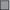) 0 ng ml^**−**1^or (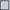) 200 ng ml^**−**1^ of *P. gingivalis*–LPS, for 2–28 d. mRNA was isolated and the levels were determined by reverse transcription over 35 polymerase chain reaction (PCR) cycles, using the appropriate primers. Polymerase chain reaction products were separated on agarose gels, examples of which are shown. The experiments were performed in duplicate and repeated on three separate occasions. Gel images were examined by densitometric analysis and the volume densities of the amplified products were normalized against glyceraldehyde-3-phosphate dehydrogenase (GAPDH) control values. Mean and standard deviation from triplicate samples were calculated to observe trends in the data. Lipopolysaccharide (LPS) was observed to have a major effect on the pattern of mRNA expression for both biglycan and decorin.

All subsequent studies investigating the effect of LPS on proteoglycan expression utilized bone cells supplemented with 200 ng ml^**−**1^ of *P. gingivalis* LPS, where effects on cell numbers and alkaline phosphatase were most marked, without being lethal to the cells. Reverse transcription–polymerase chain reaction (RT-PCR) analysis demonstrated differential gene expression in the presence and absence of LPS, as shown in Fig. 2. Densitometric analysis of the RT-PCR products obtained for biglycan and decorin, and normalization against GAPDH levels, gave a semiquantitative analysis. While this method of quantification does not provide a highly accurate value, it does allow us to identify trends and gross changes in the expression patterns observed in the presence and absence of LPS. In the absence of LPS, high expression of biglycan mRNA was observed at 2 d, which corresponded to a rapid proliferation in cell number (Fig. 2A). In the presence of LPS, biglycan mRNA was considerably reduced. At 5 and 10 d, a period that our characterization studies suggested was associated with the early deposition of a collagenous matrix within the culture (described above), biglycan mRNA expression was negligible in the absence of LPS, but substantial levels were observed in the presence of LPS. On 17, 20, and 28 d, when our characterization studies indicated expression of bone-differentiation proteins and the formation of calcium phosphate mineral-containing nodules, high levels of biglycan mRNA expression appeared to be resumed in the absence of LPS. However, the levels of expression were reduced in the presence of LPS at 17, 20, and 28 d.

Expression of decorin mRNA by cells in the absence of *P. gingivalis* LPS rose steadily at 2, 5, and 10 d, fell slightly at 17 d and then rose again during the formation of mineralizing nodules from 20 d onwards (Fig. 2B). For bone cells grown in the presence of LPS, small, and possibly debatable, changes in decorin mRNA levels were apparent on 2, 5, 17, and 20 d. However, in the presence of LPS, large reductions in the expression of decorin mRNA were observed at 10 d (associated with the synthesis of a collagen matrix) and 28 d (associated with mineral nodule formation).

### Influence of *P. gingivalis* LPS on synthesis and extracellular processing of biglycan and decorin

**Fig. 3:**
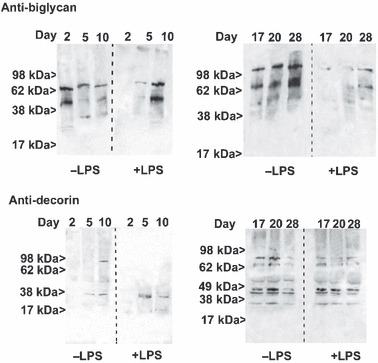
Effect of *Porphyromonas gingivalis* lipopolysaccharide (LPS) on extracellular synthesis and remodelling of biglycan and decorin present within the extracellular matrix synthesized and remodelled by the alveolar bone cells. Cells were seeded at 1 × 10^4^ cells per cm^2^, in the presence of 0 or 200 ng ml^**−**1^ of *P. gingivalis* LPS and maintained in culture for 2–28 d. Proteoglycans were extracted from the synthesized matrix by treatment with 4 M guanidinium chloride and then purified by anion-exchange chromatography. The proteoglycan-rich fractions were examined by western blot analysis using polyclonal antibodies LF106 (anti-biglycan) and LF113 (anti-decorin). Clear differences in the protein levels of biglycan and metabolic products extractable from the matrix were evident in the presence (+) and absence (−) of LPS. Difference in the higher molecular weight decorin protein bands were evident on comparing protein expression into the matrix in the presence and absence of LPS, indicating changes in the extracellular processing of this proteoglycan in the matrix. Experiments were performed in duplicate on three separate occasions. A typical western blot image is shown.

Proteoglycans were extracted from the extracellular matrix synthesized by the cells in the presence and absence of LPS. For each time-point examined, proteins were fractionated by anion-exchange chromatography and a proteoglycan-rich fraction was identified eluting at a salt concentration of 0.6–0.75 M NaCl from a Resource Q column, typical of our previously published elution profiles isolating proteoglycan from mineralized tissues (15). Western blot immunocharacterization of biglycan and decorin, and their respective processed fragments, isolated from the extracellular matrix surrounding cells incubated in the presence or absence of *P. gingivalis* LPS, is shown in Fig. 3. Immunoreactivity for biglycan was detected on day 2 for cells cultured in the absence of *P. gingivalis* LPS, with bands of approximately 62 and 49 kDa evident. Protein levels appeared to decrease during matrix maturation (5 and 10 d), with a notable loss of the 49 kDa band. These bands re-appeared during mineral deposition at 17, 20, and 28 d, with an additional band evident at approximately 98 kDa. In the presence of *P. gingivalis* LPS, the appearance of biglycan in the matrix appeared to be delayed, with protein levels notably reduced at 2 and 5 d, which then appreciably increased at 10 d. During phases when calcium phosphate mineral deposition was expected, biglycan protein levels were greatly reduced once more.

In the absence of LPS, immunodetection for decorin produced a visible immunoresponse after 5 d, by cells cultured in the absence of LPS. On day 5 a faint single band at 28 kDa was apparent, which probably represents a degradation product because the calculated molecular weight of the decorin core protein is approximately 49 kDa (14). Faint decorin bands of 28 and 78 kDa were also evident on day 10. In the presence of *P. gingivalis* LPS on day 5, there was a small increase in detectable decorin core protein. At day 10, the higher-molecular-weight band was undetectable, although the 28 kDa band was still apparent, suggesting alteration in the extracellular proteolytic processing of this proteoglycan. In the absence of *P. ginigvalis* LPS, decorin stained much more strongly at 17, 20, and 28 d, with bands evident at approximately 78, 62, 49, 42, 38, and 28 kDa. In the presence of *P. gingivalis* LPS, there was a notable reduction in the intensity of the 78- and 62-kDa decorin bands on days 17 and 20, again suggesting altered extracellular proteolytic processing. No difference was apparent for the level of decorin at 28 d. As a negative control, the immunizing peptide sequence for biglycan or decorin was premixed with the corresponding polyclonal antibody, prior to incubation with the blotted PDVF membranes. For both antibodies, immunoreactivity with the blotted proteins was completely lost, indicating that no non-specific binding of the antibody with proteins other than biglycan and decorin had occurred (data not shown).

### The effects of *P. gingivalis* LPS on GAG constituents

**Fig. 4:**
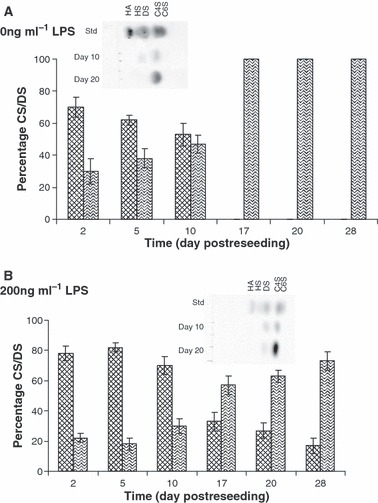
Percentage distribution of sulfated glycosaminoglycan (GAG) conjugated to proteoglycans, synthesized by the alveolar bone cells, cultured in the presence of (A) 0 and (B) 200 ng ml^**−**1^*P. gingivalis* lipopolysaccharide (LPS) for 2–28 d. Proteoglycans were isolated from the synthesized matrices by 4 M guanidinium chloride and anion-exchange chromatography. Experiments were performed in duplicate on three separate occasions. GAGs were released from protein cores by protease digestion, separated by cellulose acetate electrophoresis, and visualized by Alcian blue staining. GAGs were semiquantified by densitometric analysis, where volume densities were compared with GAG standards applied at a known concentration. Chondroitin sulfate (CS) (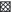) and dermatan sulfate (DS) (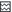) were both identified within the early stages of culture. In the presence of LPS, DS continued to persist within the cellular matrix up to 28 d. Inserts show examples obtained for the separation of GAG associated with bone cultures at 10 and 20 d. C4S, chondroitin 4-sulphate; C6S, chondroitin 6-sulphate; HA, hyaluronan; HS, heparin sulphate; Std, standard.

Examples showing the separation (by cellulose acetate electrophoresis) of the constituent GAGs, conjugated to the proteoglycan core proteins and synthesized in the presence and absence of LPS, are shown in Fig. 4. Glycosaminoglycans were identified by their electrophoretic mobility and by their susceptibility to chondroitinases. The faster migrating band was susceptible to both chondroitinases AC and ABC, identifying this material as CS. The medium migrating band was susceptible to chondroitinase ABC digestion only, thus confirming the identity of this band as DS. The slowest migrating band was identified as hyaluronan, but its presence may be attributable to serum added to the culture medium and not necessarily derived from the alveolar bone cells. Hence, the levels of GAGs, calculated from the volume densities of the Alcian blue-stained bands, were expressed as percentages of DS and CS (Fig. 4). For alveolar bone cells cultured in the absence of *P. gingivalis* LPS during phases associated with cell growth (2 and 5 d), both DS and CS were detected, with higher levels of DS present (DS: 70% at 2 d; 62% at 5 d), compared with CS. During the matrix-formation phase (10 d), DS represented 53% of the sulfated GAG. Following exposure to *P. gingivalis* LPS, during culture periods associated with cell proliferation and matrix formation, the percentage ratio of DS to CS was noticeably increased compared with the no-LPS control. During the mineralization phase (17–28 d), CS was the only sulfated GAG detected when alveolar bone cells were cultured in the absence of LPS. However, in the presence of the LPS, DS was still apparent within the matrix during periods where mineral deposition was expected.

## Discussion

Pathogenic agents that hamper the ability of the host tissue cells to initiate bone repair during periodontal disease are important considerations in continued disease progression. Bone repair may be viewed as a series of distinct, but overlapping, stages that can be recapitulated *in vitro.* The influence of *P. gingivalis* LPS on the alveolar bone cells is thus considered in terms of developmental stages related to cell proliferation, osteoid production (coinciding with collagen synthesis), and mineral deposition (coinciding with bone sialoprotein, osteocalcin synthesis, and detection of calcium phosphate mineral by von Kossa staining).

Our results demonstrated that *P. gingivalis* LPS enhanced the proliferation of alveolar bone cells, but appeared to impede the formation of the osteoblast phenotype, as evidenced by reduced alkaline phosphatase activity. These results contribute to other related, albeit conflicting, published findings. A similar decrease in alkaline phosphatase activity has been demonstrated using rat calvarial cells cultured in the presence of *P. gingivalis* LPS (33, 41), *Prevotella intermedia* LPS (42) or sonicated extracts of *P. gingivalis* (5). These studies also demonstrated a concomitant decrease in mineral deposition, as established by reduced bone nodule formation and reduced incorporation of calcium or phosphate.

These results do contradict the results of studies published previously. For instance, sonicated extracts of *P. gingivalis* had no effect on the alkaline phosphatase activity of MC3T3-E1, a pre-osteoblast-like cell line (43); and *P. gingivalis* LPS had no effect on the cell number (as determined by DNA content) of cultures derived from the periosteum of calvarial bone (33). These contradictions may reflect different biological responses relative to the source and progenitor status of the cells analysed. The cells analysed from alveolar bone chips derive from progenitor cells from periodontal ligament lining the bone surface (including Sharpey’s fibres), or marrow cavities exposed as a consequence of preparing small bone chips. The cell model system used in the present study is thus highly relevant for understanding periodontal disease because these progenitor populations represent the two main sources of cells recruited during attempts at periodontal tissue repair. Of note, studies characterizing stem/progenitor cell populations of dental tissues have suggested that the periodontal ligament contains a unique population of postnatal stem cells distinct from bone marrow (36). Furthermore, *P. gingivalis* LPS has been shown to stimulate cell proliferation in human periodontal ligament cells (44). The source and preparation of the pathogenic agent should also be considered in regard to the diversity of the published findings. Different cellular responses regarding the alkaline phosphatase activity of MC3T3-E1 cells have been reported for sonicated extracts of *P. gingivalis*, *P. intermedia*, and *Aggregatibacter (Actinobacillus) actinomycetemcomitans* (43). Likewise, *P. intermedia* and *A. actinomycetemcomitans* produce different apoptopic responses on osteoblast-like cell lines, including MG63 (45). The structure of LPS traditionally consists of three main domains and chemical differences in the residue composition of the inner oligosaccharide core and of the length and phosphorylation of the lipid A component have been identified to exist on comparison of the LPS from all these periodontal pathogens with that of *E. coli* (46). Such differences have been suggested to be responsible for the differences in the endotoxic potential observed for these different pathogens (46). Differential cellular effects were obtained when comparing sonicated bacterial extracts with purified LPS. Our study extensively purified LPS, confirming the removal of protein and DNA that are present in commercial sources of LPS from *E. coli* and have the potential to exhibit an effect on cellular activity.

A major focus of the present study was the influence of *P. gingivalis* LPS on the synthesis of biglycan and decorin (which have roles in cellular signalling, matrix formation, and mineralization) within the matrix (18). During periods within the culture associated with cell proliferation (1–5 d), mRNA and protein expression of biglycan was initially high and then fell, prior to collagen deposition. In the presence of *P. gingivalis* LPS, biglycan expression appeared to be delayed, absent on day 2, high on day 5, and continuing to day 10. The expression of biglycan during early osteogenesis is a major consideration in the bone repair process because it is increasingly recognized to provide a role in regulating cell activity. Calvarial-derived osteoblasts from biglycan-deficient mice have reduced the differentiation capability and reduced the responsiveness to BMP-4 (47). Biglycan has also been described as a positive modulator of BMP-2 osteoblast differentiation (48) and has an ability to bind growth factors, including TGF-β and TNF-α (23–25), sequestering such molecules to the matrix and thus indirectly influencing cellular activity.

Within our culture system of alveolar bone cells, collagen deposition was first observed around day 7. Decorin, but not biglycan, has been shown to bind to collagen and influence collagen fibril formation (19, 20, 22), which ultimately provides the provisional matrix for mineral deposition. In the presence of *P. gingivalis* LPS, this study reported increased levels of decorin on day 5, just prior to collagen synthesis. However, on day 10, when collagen fibril formation was expected, decorin levels were reduced compared with the no-LPS control. In addition, extracellular proteolytic processing of decorin at this time point appeared to be altered, with the loss of the higher-molecular-weight decorin band. The altered decorin expression would ultimately influence collagen fibril assembly, producing a matrix inadequate for proper mineralization. Studies have shown that DS-substituted decorin, isolated from soft connective tissues, retards the rate of collagen fibrillogenesis (19, 20). Conversely, CS-substituted decorin, isolated from cartilage or produced through recombinant techniques, appeared to promote collagen fibrillogenesis (19, 20, 22). In the presence of *P. gingivalis* LPS, DS-substituted proteoglycan persisted within the matrix, which would present an additional effect on collagen fibrillogenesis during osteoid formation.

Whilst not quantifiably established in this study, other studies have demonstrated that LPS affects mineral deposition by bone cells *in vitro* (5, 33). Both decorin and biglycan have been proposed to have roles in regulating the deposition of mineral in bone. Pertinent to this study, *in vitro* studies have shown that the inclusion of DS–biglycan or CS–bone proteoglycans into a gelatin matrix, saturated in calcium and phosphate ions, promoted hydroxyapatite deposition, but mineral deposition was inhibited in gels containing DS–decorin (26). The continued presence of DS-substituted decorin (or a process product) in the matrix of alveolar bone cells cultured in the presence of *P. gingivalis* LPS would therefore have a further inhibitory effect on the formation of mineralized bone nodules. In addition, the present study demonstrated reduced biglycan levels at time points associated with mineral deposition. Biglycan is a proteoglycan which Boskey and co-workers suggested played a more significant role in the regulation of mineralization than decorin (26).

To conclude, this study has identified that *P. gingivalis* LPS is capable of increasing the proliferation of progenitor cell populations derived from alveolar bone, delaying their subsequent differentiation (as judged by reduced alkaline phosphatase activity and altered expression of decorin and biglycan) with potential influences on alveolar bone cell activity and matrix formation. The influence of LPS on the synthesis of matrix proteins, such as biglycan and decorin, therefore presents one, albeit significant, consequence of the pathogenic action of *P. gingivalis* in the progression of periodontal disease. Raised levels of decorin and biglycan in the gingival crevicular fluid (GCF) have been proposed as potential markers of alveolar bone destruction, during periodontal disease and peri-implantitis (49, 50). The knowledge that the expression of decorin and biglycan by alveolar bone cells, derived from periodontal ligament and bone marrow sources, is reduced in the presence of LPS, confirms that the decorin and biglycan observed in the GCF during these clinical situations derives from the degradation of the matrix, rather than from attempts of the tissue to undergo repair.
